# Specific triacylglycerol, diacylglycerol, and lyso-phosphatidylcholine species for the prediction of type 2 diabetes: a ~ 16-year prospective study in Chinese

**DOI:** 10.1186/s12933-022-01677-4

**Published:** 2022-11-05

**Authors:** Junda Zhong, Chloe Y. Y. Cheung, Xiuli Su, Chi-Ho Lee, Yi Ru, Carol H. Y. Fong, Yan Liu, Cynthia K. Y. Cheung, Karen S. L. Lam, Zongwei Cai, Aimin Xu

**Affiliations:** 1 Department of Medicine, The University of Hong Kong, Hong Kong, China; 2 State Key Laboratory of Pharmaceutical Biotechnology, The University of Hong Kong, Hong Kong, China; 3 State Key Laboratory of Environmental and Biological Analysis, Department of Chemistry, Hong Kong Baptist University, Hong Kong, China; 4 Department of Pharmacology & Pharmacy, The University of Hong Kong, Hong Kong, China

**Keywords:** Type 2 diabetes, Lipidomic study, Prediction model, Glucose-stimulated insulin secretion, Triacylglycerol, Diacylglycerol, Lyso-phosphatidylcholine

## Abstract

**Background:**

Bioactive lipids play an important role in insulin secretion and sensitivity, contributing to the pathophysiology of type 2 diabetes (T2D). This study aimed to identify novel lipid species associated with incident T2D in a nested case–control study within a long-term prospective Chinese community-based cohort with a median follow-up of ~ 16 years.

**Methods:**

Plasma samples from 196 incident T2D cases and 196 age- and sex-matched non-T2D controls recruited from the Hong Kong Cardiovascular Risk Factor Prevalence Study (CRISPS) were first analyzed using untargeted lipidomics. Potential predictive lipid species selected by the Boruta analysis were then verified by targeted lipidomics. The associations between these lipid species and incident T2D were assessed. Effects of novel lipid species on insulin secretion in mouse islets were investigated.

**Results:**

Boruta analysis identified 16 potential lipid species. After adjustment for body mass index (BMI), triacylglycerol/high-density lipoprotein (TG/HDL) ratio and the presence of prediabetes, triacylglycerol (TG) 12:0_18:2_22:6, TG 16:0_11:1_18:2, TG 49:0, TG 51:1 and diacylglycerol (DG) 18:2_22:6 were independently associated with increased T2D risk, whereas lyso-phosphatidylcholine (LPC) O-16:0, LPC P-16:0, LPC O-18:0 and LPC 18:1 were independently associated with decreased T2D risk. Addition of the identified lipid species to the clinical prediction model, comprised of BMI, TG/HDL ratio and the presence of prediabetes, achieved a 3.8% improvement in the area under the receiver operating characteristics curve (AUROC) (p = 0.0026). Further functional study revealed that, LPC O-16:0 and LPC O-18:0 significantly potentiated glucose induced insulin secretion (GSIS) in a dose-dependent manner, whereas neither DG 18:2_22:6 nor TG 12:0_18:2_22:6 had any effect on GSIS.

**Conclusions:**

Addition of the lipid species substantially improved the prediction of T2D beyond the model based on clinical risk factors. Decreased levels of LPC O-16:0 and LPC O-18:0 may contribute to the development of T2D via reduced insulin secretion.

**Supplementary Information:**

The online version contains supplementary material available at 10.1186/s12933-022-01677-4.

## Introduction

The global epidemic of type 2 diabetes (T2D) has been growing faster than expected over the last two decades, rising from over 150 million cases in 2000 to more than 530 million cases in 2021 [1]. This number was projected to further increase to more than 640 million in 2030 and over 780 million in 2045 [1]. To reduce the global burden of diabetes, it is important to discover novel biomarkers both for the early prediction of T2D onset and a comprehensive understanding of the pathophysiological changes preceding the disease.

Bioactive lipids play an important role in the regulation of glucose homeostasis [2, 3]. It is well established that the dysregulation of these lipids leads to the development of T2D. The overflow of lipids leads to lipotoxicity, causing impaired insulin signaling in skeletal muscle, altered secretion of hepatokines in the liver [4], and pancreatic β-cell dysfunction or cell loss [5]. On the other hand, certain lipids have shown a beneficial effect on insulin secretion [6]. Traditional indicators of lipid metabolism measured in the blood, such as triacylglycerols (TGs), high-density lipoprotein (HDL), low-density lipoprotein (LDL), TG/HDL ratio and triglyceride-glucose (TyG) index, have been identified as predictors of T2D in previous studies [7]. However, the plasma lipidome comprises thousands of lipid species. Clinical assays of blood lipids failed to address the complexity and diversity of lipid species. With the recent advances in lipidomics, the identification resolution and coverage of lipid species have been unprecedentedly enhanced and expanded.

Accumulative studies have investigated the association between human plasma lipidome and the pathogenesis of T2D [8–14]. Lipid species identified by lipidomics have been shown to be superior over the traditional lipotoxicity indicators in the prediction of T2D development. Prospective lipidomic studies in Asians assessing the link between lipid species and the risk of T2D have recently emerged [8, 9, 15–18]. However, most of them have relatively short follow-up duration and have adopted the targeted approach which has limited ability to provide a comprehensive investigation of novel predictive lipid species. Here we used a cohort of Chinese residents in Hong Kong (mean age ± standard deviation: 51.13 ± 11.57 years) with a long follow-up period of almost 16 years to identify novel lipid species that are associated with incident T2D.

In the current study, we aimed to: (i) discover novel lipid species associated with incident T2D by performing an untargeted lipidomic analysis in a nested case–control study within a prospective population-based cohort; (ii) evaluate the improvement of prediction for incident T2D provided by the identified lipid species; and (iii) investigate the effect of the identified lipid species on insulin secretion.

## Methods

### Study population

A nested case–control study was performed in the Hong Kong Cardiovascular Risk Factor Prevalence Study (CRISPS). CRISPS is a prospective, population-based longitudinal cohort designed to study the development of cardiovascular risk factors, including diabetes, in Hong Kong from 1995 to 2018. Details of the CRISPS cohort were previously described elsewhere [19, 20]. Briefly, in 1995–1996 (CRISPS-1), 2895 Hong Kong Chinese, aged 25–74, were randomly recruited from the general population of Hong Kong to undergo a comprehensive assessment. Subjects were followed up in CRISPS-2 (2000–2004), CRISPS-3 (2005–2008), CRISPS-4 (2010–2012) and CRISPS-5 (2016–2018). All subjects had blood taken at each visit after an overnight fast of at least 10 h. Details of anthropometric measurements and methods for the measurement of biochemical parameters were described previously [20]. Hypertension was defined as blood pressure ≥ 140/90 mmHg or the use of antihypertensive medications. A 75 g oral glucose tolerance test (OGTT) was performed in all except those taking antidiabetic medications. T2D was defined as fasting plasma glucose ≥ 7.0 mmol/l or 2-h plasma glucose ≥ 11.1 mmol/l during OGTT, or on antidiabetic medications, according to the World Health Organization (WHO) 1998 diagnostic criteria [21]. Impaired fasting glucose (IFG) was defined as fasting plasma glucose 6.1–6.9 mmol/L and 2-h plasma glucose < 7.8 mmol/L. Impaired glucose tolerance (IGT) was defined as fasting plasma glucose < 7 mmol/L and 2-h plasma glucose ≥ 7.8 and < 11.1 mmol/L [21]. Prediabetes was defined as the presence of IFG and/or IGT. TG/HDL ratio was calculated by the following formula: TG/HDL ratio = TG (mg/dl)/HDL (mg/dl). Homeostatic model assessment of insulin resistance (HOMA-IR) was calculated using the following formula: HOMA-IR = fasting insulin (µU/l) × fasting glucose (mmol/l)/22.5 [22]. Homeostatic model assessment of β-cell function (HOMA-β) was calculated using the following formula: HOMA-β = [20 × fasting insulin (µU/ml)]/[fasting glucose (mmol/l) − 3.5] [22]. All participants had given written informed consent. Ethics approval was obtained from the Institutional Review Board of the University of Hong Kong/Hospital Authority, Hong Kong West Cluster. The lipidomic analysis was performed on the stored plasma samples collected at CRISPS-2, therefore, this visit was considered as the baseline of the current study.

### Clinical outcome

Subjects who were free from T2D at CRISPS-2 (i.e., baseline of the current study) were followed for their glycaemic status at the subsequent visits. Incident T2D cases included those who had developed T2D when assessed at the CRISPS-3, CRISPS-4 or CRISPS-5 visits. The persistent non-T2D controls were those who remained free from T2D at CRISPS-5. In this prospective nested case–control study, the eligible baseline population was limited to those who did not have T2D at baseline and with plasma samples available for the lipidomic analysis. Subjects who were on lipid-lowering drugs at baseline were further excluded to minimize the possible drug effects on lipid metabolism. Each of the 196 incident T2D cases who fulfilled the inclusion criteria was age- and sex-matched with a non-T2D control who remained free of T2D at the end of the follow-up (CRISPS-5) using the propensity score matching method.

### Chemicals

All lipid standards were purchased from Avanti Polar Lipids except for triacylglycerol (TG) 15:0_15:0_15:0, lyso-phosphatidylcholine (LPC) O-16:0, LPC O-18:0 and palmitic acid. TG 15:0_15:0_15:0 and palmitic acid were obtained from Sigma-Aldrich. DG 18:2_22:6 and TG 12:0_18:2_22:6 were synthesized using a method described by Halldorsson et al. [23], as detailed in Additional file 1: Methods. LPC O-16:0 and LPC O-18:0 were purchased from Cayman Chemical (Michigan, USA) and diluted in phosphate-buffered saline (PBS) at a stock concentration of 5 mmol/l prior to the analysis. Palmitic acid was dissolved in ethanol at a stock concentration of 20 mmol/l. DG 18:2_22:6 and TG 12:0_18:2_22:6 were dissolved in dimethyl sulfoxide at a stock concentration of 20 mmol/l.

### Lipidomic analyses

Lipids were extracted from 40 μL of plasma based on a modified method of Matyash et al. [24]. Untargeted lipidomic analysis was performed on a Thermo UltiMate 3000 ultra-high performance liquid chromatography tandem mass spectrometry (UHPLC) system coupled to a Thermo Orbitrap Fusion mass spectrometer [25]. Targeted lipidomic analysis of the selected lipids was performed on a Vanquish™ UHPLC Systems coupled to TSQ Altis™ Triple Quadrupole Mass Spectrometer (Thermo Scientific, USA). More details are provided in Additional file 1: Methods.

### Mouse islet isolation

Mouse pancreatic islets were isolated, cultured overnight and picked under a microscope for the glucose-stimulated insulin secretion assay (GSIS). The detail of islet isolation is described in Additional file 1: Methods. All animal experimental protocols were approved by the Animal Ethics Committee of The University of Hong Kong.

### Glucose-stimulated insulin secretion assay

The isolated mouse islets were fasted for 120 min with glucose-free Krebs buffer containing 10 mmol/l HEPES, 129 mmol/l NaCl, 4.8 mmol/l KCl, 1.2 mmol/l MgSO_4_∙7H_2_O, 1.2 mmol/l KH_2_PO_4_, 2.5 mmol/l CaCl_2_∙2H_2_O, 5 mmol/l NaHCO_3_ and 0.1% fatty acid-free bovine serum albumin (pH 7.4). Next, the islets were incubated with Krebs buffer containing 2 mmol/l glucose, 20 mmol/l glucose, or 20 mmol/l glucose with 10 μmol/l or 50 μmol/l of LPC O-16:0, LPC O-18:0, DG 18:2_22:6, TG 12:0_18:2_22:6, and palmitic acid for another 30 min (n = 9 in each experimental group with 10 islets per well). The buffer samples were then collected for the measurement of insulin level using an in-house mouse high-sensitive insulin ELISA kit (ImmunoDiagnostics Limited, The University of Hong Kong).

### Statistical analyses

All analyses were conducted in R v4.3.0. Normally distributed data were presented as means ± standard deviation. Non-normally distributed data (determined by Kolmogorov–Smirnov test) were transformed by natural logarithm to near normality before the analysis and presented as median (interquartile). Missing data were rare (Table 1) and median imputation was used for the missing values. Student *t*-tests were used to compare continuous variables while Pearson χ^2^ tests and Fisher’s exact tests were applied to compare categorical variables at baseline examination between the incident case and non-T2D control groups.

**Table 1:** Baseline characteristics of study participants according to glycaemic status on long-term follow-up

	Incident T2D n = 196	Persistent non-T2D n = 196	Missing data (n)	p-value^b^
Age (years)	51.7 ± 9.8	52.1 ± 9.8	0	0.719
Male (%)	48.5	48.0	0	1.00
BMI (kg/m^2^)	25.8 ± 3.4	24.1 ± 2.9	0	< 0.001
Fasting glucose (mmol/l)	5.40 ± 0.55	5.06 ± 0.45	0	< 0.001
2-h glucose (mmol/l)	8.09 ± 1.69	6.51 ± 1.56	0	< 0.001
Prediabetes (%)	60.20	27.55	0	< 0.001
HOMA-IR^a^	2.3 (1.5-3.1)	1.6 (1.1-2.2)	1	< 0.001
HOMA-β^a^	100.0 (66.5–156.1)	98.7 (72.8–130)	1	0.387
TC (mmol/l)^a^	5.40 (4.80–5.90)	5.30 (4.80–5.80)	2	0.454
TG (mmol/l)^a^	1.35 (1.00–2.00)	1.00 (0.80–1.43)	2	< 0.001
LDL-C (mmol/l)^a^	3.40 (2.90–3.90)	3.40 (2.90–3.80)	5	0.971
HDL-C (mmol/l)^a^	1.24 (1.03–1.45)	1.30 (1.16–1.63)	2	< 0.001
TG/HDL ratio	2.48 (1.66–4.17)	1.70 (1.15–2.56)	2	< 0.001
Creatinine (μmol/l)^a^	80.0 (67.00–96.00)	79.0 (62.75–94.00)	1	0.232
Hypertension (%)	30.1	21.9	0	0.066
CVD (%)	3.1	0.5	0	0.122

Data are presented as mean ± SD or median (interquartile range)

*T2D* type 2 diabetes, *BMI* body mass index, *HOMA-IR* homeostasis model assessment of insulin resistance, *HOMA-β* homeostasis model assessment of β-cell function, *TC* total cholesterol, *TG* triacylglycerol, *LDL-C* low-density lipoprotein-cholesterol, *HDL-C*, high-density lipoprotein cholesterol, *TG/HDL ratio* triacylglycerol to high-density lipoprotein cholesterol ratio, *CVD* cardiovascular disease

^a^Natural log-transformed before analysis

^b^*P* values of plasma indicators were obtained from independent samples tests or Pearson tests for the binary variables

Lipid species levels were transformed by natural logarithm to near normality before the analysis. To investigate the connection within the lipid species, the R package “weighted gene co-expression network analysis (WGCNA)” was used to determine modules of highly interconnected lipid species. It includes feature co-expression, network construction, module identification, module-phenotype correlation recognition, and key driver gene identification. Highly connected lipid species were defined in a colored module by topological overlap measure (TOM). Finally, a weighted co-expression network generated by WGCNA was visualized by Cytoscape (version 3.7.1).

Boruta analysis was applied to select features that were most important for the prediction of T2D. The algorithm generates randomly shuffled copies of lipid species concentrations (shadow features). Lipid species with higher Z-scores than the maximum Z-score of their shadow features were categorized either as confirmed or tentative features [26]. These lipid species were then selected and quantified using the targeted lipidomic analysis.

Stepwise model selection approach was used to identify the best model that comprised the most important clinical variables for incident T2D. Multiple conditional logistic regression analyses were then performed to assess the independent associations of the lipid species with incident T2D after adjustment for the most important clinical predictors identified. The false discovery rate (FDR) using the Benjamin-Hochberg method was employed for the correction of multiple testing. A two-sided p-value < 0.05 and an FDR-adjusted p-value (*q*-value) < 0.1 was considered statistically significant. Area under the receiver operating curves (AUROCs) was estimated to evaluate the predictive ability of the lipid species that showed statistically significant and independent associations with incident T2D. The Delong test was used for comparing the AUROCs. The improvement of the predictive ability given by the lipid species was further quantified by continuous net reclassification index (cNRI) and integrated discrimination index (IDI) [27]. A two-tailed p-value < 0.05 was considered statistically significant.

For the GSIS study, data were presented as the mean ± mean standard error (SEM) and were analyzed using student’s t-test or one-way analysis of variance with the post-hoc test. A two-sided p-value < 0.05 was considered statistically significant.

## Results

The baseline characteristics of the study participants are presented in Table 1. The median follow-up time of the 196 incident T2D cases and 196 age- and sex-matched non-T2D controls was 15.57 years (interquartile range [IQR] 9.98–16.97). As expected, subjects who had developed T2D showed significantly higher BMI, fasting glucose, 2-h glucose, the presence of prediabetes, HOMA-IR, TG, and TG/HDL ratio at baseline compared to the non-T2D controls. Based on stepwise model selection, the best clinical model predictive of incident T2D comprised of BMI, TG/HDL ratio and the presence of prediabetes (Additional file 2: Table S1).

### Lipid profiling and co-expression network

A total of 301 lipid species across 13 classes were detected from the untargeted lipidomic analysis (Additional file 2: Table S2). To understand the complex processes of lipid dysregulation preceding T2D, a lipid co-expression network was constructed using WGCNA and 13 modules were generated (Additional file 1: Fig. S1a–c and Additional file 2: Table S3). The lipid species that were not co-expressed with other lipid species were grouped in the grey module, which was ignored in further analyses. The threshold analyses and the cluster dendrogram of the network construction were shown in Additional file 1: Fig. S1a–c. After adjustment for BMI, TG/HDL ratio and the presence of prediabetes, the eigenvalues of the blue, pink, green-yellow, black, and turquoise modules containing lipid classes of TG, diacylglycerol (DG), phosphatidylcholine (PC) and phosphatidylethanolamine (PE), were positively correlated with incident T2D (p < 0.05), whereas the red, green, yellow and brown modules comprising of the ether-PCs, ether-PEs, sphingomyelins (SMs), acyl-LPCs and ether-LPCs were negatively associated with T2D risk (Additional file 1: Fig. S1d, e). An interaction network generated by Cytoscape 3.8.2 based on these modules was shown in Additional file 1: Fig. S1f. Lipid species with higher connectivity were closer to the center of the network. In the multiple conditional logistic regression analysis based on the quartiles of module eigenvalues, the highest quartile of module blue (mainly TGs and PCs) was positively associated with T2D compared to the lowest quartile (OR [95% CI] 2.18 [1.13–4.22]; *p* for trend = 0.001) after adjustments for BMI, TG/HDL ratio and the presence of prediabetes. On the other hand, module brown (mainly acyl-LPCs and ether-LPCs) was inversely associated with T2D (lowest OR [95% CI] 0.21 [0.09–0.47]; p for trend = 0.035) (Table 2).

**Table 2:** Associations between weighted gene co-expression network analysis module eigenvalues and incident type 2 diabetes

Modules	Q1	Q2 (ORs [95% CI])	Q3 (ORs [95% CI])	Q4 (ORs [95% CI])	*P*_trend_
Blue: TGs, DGs and PCs with a 22:6 fatty acid and other LCFA; number of lipid species = 35					
	1	1.03(0.51–2.07)	1.61(0.81–3.20)	2.18(1.13–4.22)	**0.012**
Red: ether-PCs and ether-PEs with an unsaturated-LCFA; number of lipid species = 16					
	1	0.74(0.38–1.44)	0.99(0.47–2.09)	1.08(0.52–2.26)	0.700
Green: ether-PCs with a saturated-LCFA; number of lipid species = 18					
	1	1.76(0.93–3.32)	1.25(0.63–2.48)	1.21(0.53–2.77)	0.322
Yellow: SMs with LCFA; number of lipid species = 25					
	1	1.64(0.81–3.30)	1.28(0.62–2.67)	1.39(0.64–3.02)	0.457
Pink: TGs with at least one 18:2 FA; number of lipid species = 13					
	1	0.88(0.44–1.73)	0.84(0.42–1.68)	1.46(0.75–2.84)	0.121
Green-yellow: DGs with LCFA; number of lipid species = 10					
	1	0.66(0.32–1.36)	0.80(0.38–1.69)	0.52(0.22–1.27)	0.709
Black: PEs with a saturated-LCFA and an unsaturated-LCFA; number of lipid species = 14					
	1	1.47(0.78–2.77)	1.64(0.83–3.24)	1.32(0.62–2.82)	0.073
Turquoise: TGs containing LCFA with less than 2 double bonds and PCs with LCFA; number of lipid species = 47					
	1	0.63(0.32–1.23)	0.74(0.36–1.52)	1.03(0.48–2.21)	0.209
Brown: LPCs and ether LPCs containing LCFA and VLCFA; number of lipid species = 30					
	1	0.64(0.33–1.25)	0.21(0.09–0.47)	0.55(0.27–1.12)	**0.035**

The results of the modules significantly correlated to incident type 2 diabetes were shown. Boldface type indicates p for trend < 0.05 for the association with type 2 diabetes. The model is adjusted for BMI, TG/HDL ratio and prediabetes

*OR* odds ratio*, BMI* body mass index*, TG* triacylglycerol, *HDL* high-density lipoprotein, *LCFA* long-chain fatty acid, *VLCFA* very-long-chain fatty acid, *DG* diacylglycerol, *PC* phosphatidylcholine, *PE* phosphatidylethanolamine, *LPC* lyso-phosphatidylcholine, *Cer* ceramide, *SM* sphingomyelin

### Lipid species selection

Boruta analysis was used to select the most important lipid species for the prediction of T2D. The levels of importance of the lipid species were compared with the shadow-max value and 16 confirmed lipid species were identified as potential predictors of incident T2D (Additional file 2: Table S4 and Additional file 1: Fig. S2). Among these, 11 lipid species were also highlighted in the interaction network analysis (Additional file 1: Fig. S1f). These 16 selected lipid species, including 4 PCs, 5 TGs, 2 DGs, 4 LPCs and one PE, were then carried forward for absolute quantification using the targeted lipidomic analysis.

### Lipid species and the risk of T2D

All of the selected lipid species quantified by the targeted lipidomic analysis, except DG 18:0_18:0, were significantly different between the incident T2D cases and non-T2D controls (all unadjusted p < 0.01; *q*-value < 0.10) (Additional file 1: Fig. S3). Lipid species of the same category were highly correlated and the LPCs were inversely correlated with DGs as well as TGs (Additional file 1: Fig. S4).

Multiple conditional logistic regression analysis was adopted to identify the independent lipid predictors for incident T2D (Table 3). After adjustment for BMI, TG/HDL ratio, and the presence of prediabetes, TG 12:0_18:2_22:6, TG 16:0_11:1_18:2, TG 49:0, TG 51:1 and DG 18:2_22:6 were shown to be positively associated with incident T2D, and LPC O-16:0, LPC P-16:0, LPC O-18:0 and LPC 18:1 were inversely associated with incident T2D (p < 0.05, *q*-value < 0.10) (Table 3). Recognizing the potential collinearity between TG and TG/HDL ratio, we replaced TG/HDL ratio with TG in the analysis and the result remained similar.

**Table 3:** Associations between lipid species and incident T2D

	OR (95% CI)	p-value*	*q*-value*
TG 12:0_18:2_22:6	**1.24(1.06–1.44)**	**0.006**	**0.032**
TG 16:0_11:1_18:2	**1.25(1.04–1.49)**	**0.017**	**0.045**
TG 48:0	1.12(0.93–1.34)	0.231	0.334
TG 49:0	**1.28(1.02–1.62)**	**0.034**	**0.075**
TG 51:1	**1.27(1.00–1.61)**	**0.047**	**0.083**
DG 18:0_18:0	1.06(0.73–1.52)	0.774	0.774
DG 18:2_22:6	**1.36(1.10–1.68)**	**0.005**	**0.032**
PC O-16:1_18:1	0.70(0.27–1.80)	0.462	0.528
PC O-16:1_18:2	0.68(0.35–1. 31)	0.251	0.334
PC O-24:1_18:2	0.75(0.44–1.29)	0.299	0.368
PC O-34:1	0.51(0.18–1.49)	0.220	0.334
PE O-34:2	0.76(0.25–2.29)	0.631	0.673
LPC O-16:0	**0.69(0.52–0.91)**	**0.009**	**0.037**
LPC P-16:0	**0.62(0.45–0.86)**	**0.004**	**0.032**
LPC O-18:0	**0.69(0.51–0.93)**	**0.014**	**0.044**
LPC 18:1	**0.53(0.30–0.96)**	**0.037**	**0.075**

Lipid species with p-value < 0.05 and *q*-value < 0.1 are in bold

*OR* odds ratio*, CI* confidence interval, *BMI* body mass index*, TG/HDL ratio* triacylglycerol to high-density lipoprotein cholesterol ratio*, DG* diacylglycerol, *TG* triacylglycerol, *PC* phosphatidylcholine, *PE* phosphatidylethanolamine, *LPC* lyso-phosphatidylcholine

*Adjusted for BMI, TG/HDL ratio, and prediabetes

**Fig. 1:**
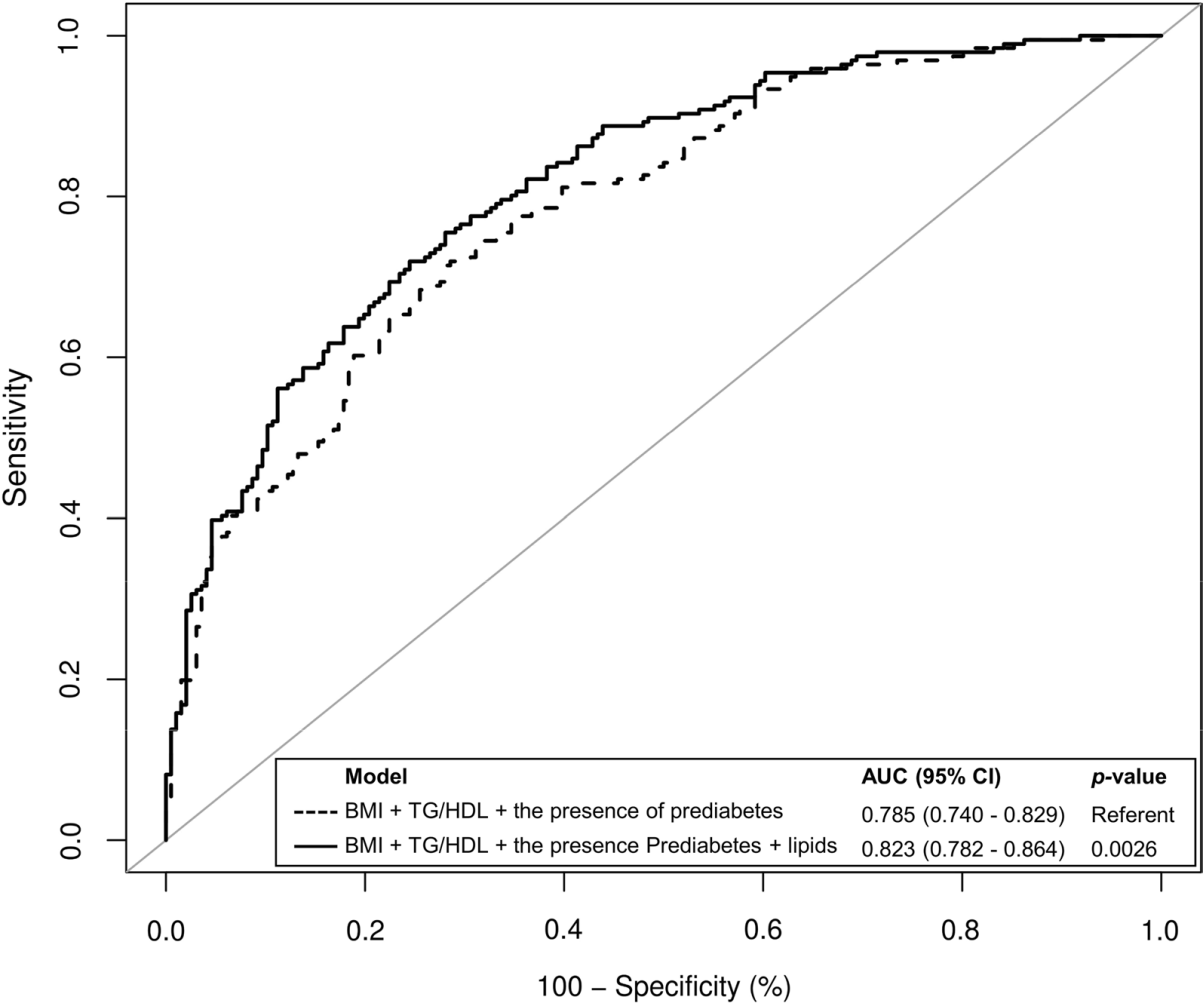
ROC curve analysis showing the AUROCs of different models for the prediction of T2D. Lipids included in the analysis were TG 12:0_18:2_22:6, TG 16:0_11:1_18:2, TG 49:0, TG 51:1, DG 18:2_22:6, LPC O-16:0, LPC P-16:0, LPC O-18:0 and LPC 18:1

Next, we assessed whether the independent lipid species showed an incremental prediction value on the development of T2D using ROC curves analyses (Fig. 1). Adding TG 12:0_18:2_22:6, TG 16:0_11:1_18:2, TG 16:0_16:0_17:0, TG 16:0_17:0_18:1, DG 18:2_22:6, LPC O-16:0, LPC P-16:0, LPC O-18:0 and LPC 18:1 to the clinical risk model significantly increased the AUROC from 0.785 to 0.823 (improved by 3.8%, p = 0.0026). The improvement was further confirmed by both cNRI (37.8%, [95% CI 18.4–57.1%], p < 0.001) and IDI (3.7% [95% CI 1.9–5.6%], p < 0.001).

### LPC O-16:0 and LPC O-18:0, but not DG 18:2_22:6 and TG 12:0_18:2_22:6, potentiated glucose-stimulated insulin secretion in mouse islets

**Fig. 2:**
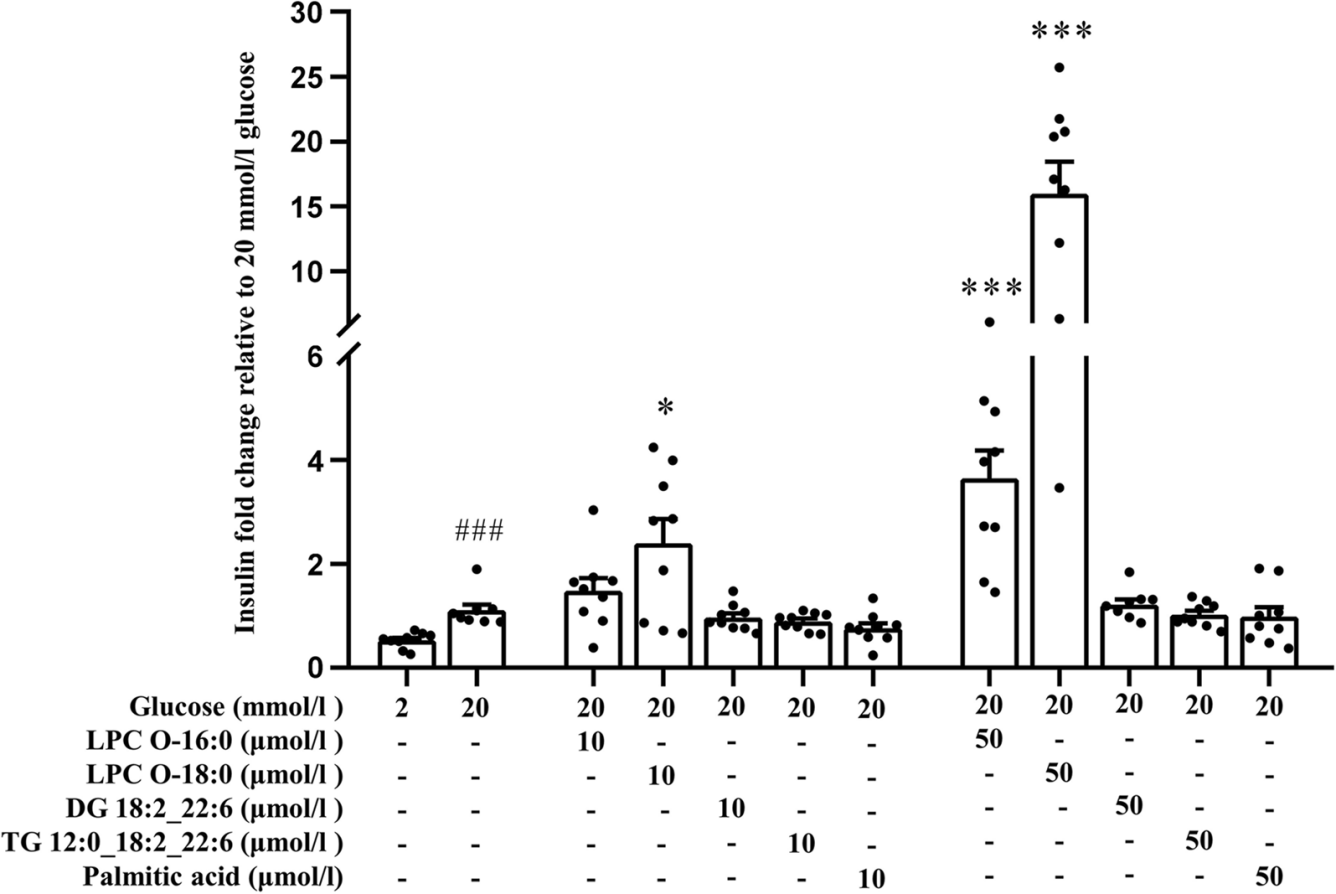
Effect of various lipid species on glucose stimulated insulin secretion in mouse islets. Mouse islets were incubated in Krebs buffer with 0 mmol/l glucose for 2 h and then treated with Krebs buffer containing 2 mmol/l glucose, 20 mmol/l glucose or 20 mmol/l glucose with increasing doses of LPC O-16:0, LPC O-18:0, DG 18:2_22:6, TG 12:0_18:2_22:6 or palmitic acid for 30 min (n = 9 in each experimental group with 10 islets per well). Insulin levels are shown as the fold change relative to the insulin secreted at 20 mmol/l glucose. Note: vehicles (ethanol or DMSO) used in the GSIS did not affect insulin secretion. ^###^p < 0.001 for 2 mmol/l glucose group vs. 20 mmol/l glucose only group; *p < 0.05, **p < 0.01 and ***p < 0.001 for test groups vs. 20 mmol/l glucose only group

The majority of East Asian patients with T2D, including Chinese, show prominent defects in insulin secretion relative to insulin resistance, and β-cell dysfunction plays a key role in the development of T2D among East Asian populations [28, 29]. We observed an inverse association of several LPCs but positive correlation of some glycerolipids with the risk of incident T2D. To interrogate whether the newly identified LPC O-16:0, LPC O-18:0, DG 18:2_22:6 and TG 12:0_18:2_22:6 modulate insulin secretion, we further investigated their possible effects on GSIS using mouse islets. Isolated mouse islets were incubated with 20 mmol/l glucose only or further supplemented with 10 µmol/l or 50 µmol/l of LPC O-16:0, LPC O-18:0, DG 18:2_22:6, TG 12:0_18:2_22:6, or palmitic acid. Under high (20 mmol/l) glucose condition, LPC O-16:0 and LPC O-18:0 significantly potentiated GSIS in a dose-dependent manner, 50 µmol/l of LPC O-16:0 and LPC O-18:0 increased GSIS by 4- and 16-fold, respectively. On the other hand, DG 18:2_22:6, TG 12:0_18:2_22:6 and palmitic acid showed no effect on GSIS (Fig. 2).

## Conclusions

In this study, we identified specific TG, DG, and LPC species that were independently associated with incident T2D in a phenotypically well-characterized Chinese population-based cohort with a long follow-up of ~ 16 years. These lipid predictors are reported in a prospective study for the first time and were able to achieve a substantial increase in the prediction of incident T2D beyond a predictive model based on the most important clinical risk factors identified in this cohort. We demonstrated their pathophysiological involvement in the onset of the disease and identified potential therapeutic targets for the treatment of T2D.

Our untargeted lipidomic analysis covered over 300 lipid species, which is larger than most of the previous studies in Asian populations [8, 9, 15–18]. The network analysis revealed a collective effect of TGs, DGs and PCs with a common docosahexaenoic acid chain (22:6), which was associated with increased T2D risks. TGs with a common linoleic acid (18:2), as well as LPCs and ether LPCs containing long-chain fatty acids and very-long-chain fatty acids showed a collective effect in the association with the onset of T2D. Our network analysis further showed that most of the lipid species independently associated with T2D were also inter-connected with a large number of other lipids that contain similar fatty acid chains, indicating that they may play a nexus role in the metabolic pathways of those lipids.

In this study, several LPCs were detected to be inversely associated with incident T2D after adjustments for BMI, TG/HDL ratio and the presence of prediabetes, the most important clinical predictors identified with the stepwise model selection approach. Among them, LPC P-16:0 and LPC 18:1 have previously been reported to be associated with T2D [11, 30], while our study is the first to show the inverse associations of LPC O-16:0 and LPC O-18:0 with incident T2D. Our findings are in agreement with previous cross-sectional studies which reported a significantly lower level of LPC O-16:0 in the skeletal muscle of T2D patients [31] and a negative association between plasma LPC O-18:0 and dysglycemia [32]. LPC O-16:0 and LPC O-18:0 are ether-linked LPCs which are also known as lyso-platelet-activating factors (lyso-PAFs). Three other lyso-PAFs, LPC O-22:0, O-24:1 and O-24:2, have been shown to be associated with a decreased risk of T2D in a prospective study conducted in the Australian Diabetes, Obesity and Lifestyle Study [33]. Lyso-PAF has long been considered as an inactive precursor of PAF, but emerging evidence suggests that lyso-PAF has its unique functions such as inhibiting the PAF-potentiated NADPH oxidase activation in neutrophils [34]. However, the role of lyso-PAFs in the pathogenesis of T2D remains unknown. Our functional study demonstrated that LPC O-16:0 and LPC O-18:0 dose-dependently potentiated GSIS in mouse islets, thereby providing evidence for their potential effect on β-cell function. It has been observed that Asians with T2D tend to have prominent impairment in β-cell function, better insulin sensitivity and lower BMI compared to Caucasians [28, 29, 35]. In this collection, our study raised the possibility that lower LPC O-16:0 and LPC O-18:0 levels may contribute to impaired insulin secretion and T2D in Chinese. This is further supported by our observation that LPC O-16:0 and LPC O-18:0 potently stimulated GSIS at the physiological range whereas the same concentrations of palmitic acid, were unable to demonstrate such an effect. Indeed, consistent with our findings, previous studies reported that palmitic acid stimulated GSIS only at a high concentration of 500 µmol/l, which is out of the physiological range [36, 37]. Such observation suggests that LPC O-16:0 and LPC O-18:0 may stimulate GSIS through a possibly unknown molecular pathway independent of the classical glycerolipid/free fatty acid pathway [38]. Further investigations are warranted to explore the signaling pathways whereby LPC O-16:0 and LPC O-18:0 potentiate GSIS and to explore their therapeutic potential for T2D.

We also detected independent associations of TG 12:0_18:2_22:6, TG 16:0_11:1_18:2, TG 49:0, TG 51:1 and DG 18:2_22:6 with increased risk of incident T2D. Our findings were consistent with previous clinical studies showing positive associations of TGs with the risk of T2D [10, 13]. In contrast, some other studies suggested that certain TGs were inversely associated with the risk of T2D [9, 11, 14]. However, these studies were conducted over relatively shorter follow-up durations, thus the cumulative detrimental effect of TGs was not prominent. Indeed, previous studies which are consistent with our findings also had a longer follow-up, with an average of 20 years [10, 13]. Our present study identified TG 12:0_18:2_22:6 and DG 18:2_22:6, which are structurally similar, as novel lipid species associated with incident T2D. Although the exact role of these two lipid species in T2D is unknown, Szendroedi et al*.* previously demonstrated a strong positive relationship of several DG lipid species (DG 16:0_18:2, 18:1_18:2, 18:2_18:2, and 18:2_18:0) comprised of a common α**-**linoleic acid (fatty acid 18:2) chain with protein kinase C theta (PKC-θ) activation in human muscle cells [39]. The activated PKC-θ would lead to a decrease in insulin-stimulated insulin receptor substrate-1 (IRS-1)/IRS-2 tyrosine phosphorylation and subsequently disturbed downstream insulin signaling, inducing insulin resistance in the muscle. In our functional analysis, we did not observe a direct effect of the newly identified TG 12:0_18:2_22:6 and DG 18:2_22:6 on GSIS. It is possible that these lipid species may affect the development of T2D via a pathway independent of insulin secretion. Previous studies have reported that the downstream products of TGs, such as acyl-CoA, DG and ceramide, impair the insulin signaling pathways thereby leading to T2D risk [40]. Further functional analyses to elucidate the potential role of these novel lipid species in T2D are warranted.

With the addition of only 9 lipid species, we were able to achieve a significantly increased predictive power compared to the clinical predictive model comprising of BMI, TG/HDL ratio and the presence of prediabetes (enhanced by 3.8%, p = 0.0026). Our results appeared to show a better improvement in prediction with the addition of lipid species compared to previous studies which commonly increased the prediction value by 1.0–3.2%, even with over 50 lipid species [10, 11].

The major strength of this study was the use of a phenotypically well-characterized prospective population-based CRISPS cohort which has a relatively long follow-up period compared to most of the population-based studies among the Asian population. Another strength was the use of the high-throughput untargeted lipidomic profiling, which is a robust, non-biased strategy for lipidomic depiction and discovery of novel biomarkers. On the other hand, targeted lipidomic analyses have higher sensitivity, specificity, and quantification ability, which is applicable to known compounds. Merging of untargeted and targeted analyses provides an alternative way to combine the advantages of novel biomarker discovery and accurate quantification. Given the enormous complexities of the human plasma lipidome, UHPLC-mass spectrometry based-lipidomics is the preferred method for in-depth studies of lipid-related pathological mechanisms and the identification of predictive biomarkers for diabetes. Moreover, the employment of untargeted lipidomics, which has provided comprehensive coverage of various categories of lipids, has allowed us to analyze the interactions among lipids using WGCNA and select potential factors from a large pool of lipids using a machine-learning method.

We acknowledge that the limitations of our study included the relatively small sample size and lack of an external validation cohort. Furthermore, hemoglobin A1c (HbA_1c_) was not evaluated in our cohort because the 1998 WHO diagnostic criteria was employed in CRISPS-2 (2000–2004), when the baseline data for the current study were collected. Nonetheless, recent studies showed that HbA_1c_ only added 0.5% to the prevalence of diabetes diagnosed by OGTT in the Chinese population [41]. Finally, all the participants were of Chinese ancestry, and hence our findings may not be generalizable to other populations.

In conclusion, our results provided novel insights into the underlying mechanisms of lipid species in the development of T2D. We discovered several novel lipid species associated with the pathophysiological changes before T2D onset. The identified lipid species substantially increased the predictive value beyond the traditional risk factors. Further validations in independent prospective population-based cohorts are required to confirm our findings. Future functional studies to elucidate the mechanistic pathways of the lipid species in T2D are warranted.

## Supplementary Information

**Additional file 1. **Methods. **Figure S1.** WGCNA of the lipid species profile. **Figure S2.** Boruta analysis result. **Figure S3.** Violin Plot of the Targeted Lipidomics. **Figure S4.** Correlation among identified lipid species.

**Additional file 2: Table S1.** Stepwise model selection coefficients and Wald-Z tests. **Table S2.** List of measured lipid species. **Table S3.** Weighted gene co-expression network analysis module components. **Table S4.** Boruta analysis result.

## Data Availability

The datasets generated during and/or analysed during the current study are available from the corresponding author on reasonable request.
