# Supplementary material for: Specific triacylglycerol, diacylglycerol, and lyso-phosphatidylcholine species for the prediction of type 2 diabetes: a ~ 16-year prospective study in Chinese
Source: Cardiovasc Diabetol. 2022 Nov 5;21:234. doi: 10.1186/s12933-022-01677-4 (PMC9637304; doi:10.1186/s12933-022-01677-4)

**Additional file 1**

**METHODS**

**Lipidomic profiling**

Lipid were extracted from 40 μl of plasma using 300 μl of methanol containing internal standards including triacylglycerol (TG) 15:0_15:0_15:0, lyso-phosphatidylcholine (LPC) 19:0_0:0, sphingomyelin (SM) d18:1_12:0, ceramide (Cer) d18:1_17:0, phosphatidylcholine (PC) 19:0_19:0 and Glucosyl C12 Cer d18:1_12:0, based on a modified method of Matyash et al (1). After vortex, 1 ml of methyl tert-butyl ether (MTBE) was added to the mixture. After another vortex for 1 min, 0.25 ml of water was added, followed by another vortex for 1 min. Then the samples were incubated for 10 min on ice and centrifuged for 10 min at 4℃. The upper organic phase was collected and freeze-dried. The extracted lipids were reconstituted with 60 μl of acetonitrile/isopropanol/water (65:30:5, v/v/v) for liquid chromatography-mass spectrometry (LC-MS) analysis. Quality control (QC) samples were prepared by mixing an equal volume of aliquots from each plasma sample and were processed as described above. All samples were randomized during sample preparation.

Lipidomics analysis was performed on a Thermo UltiMate 3000 Ultra-High Performance Liquid Chromatography (UHPLC) system coupled to a Thermo Orbitrap Fusion mass spectrometer as described previously (2). All the samples were randomized during LC-MS analysis to minimize systematic bias. QC samples were analyzed after every 10 injections of clinical samples to assess the reproducibility of the LC-MS analytical system.

The lipidomic ddMS2 data were processed by Thermo Lipid Search software, including peak picking, peak alignment and lipid identification. The mass tolerance for precursors and fragment ions was set as 10 ppm and 20 ppm respectively. Lipid species were identified by matching the precursor ions and corresponding product ions to the lipid database. Lipids were annotated at the species and molecular species levels (3). Species level annotation was conducted based on the measurements of accurate mass, isotopic pattern and characteristic fragmentation of lipid classes using a high-resolution mass spectrometer. Molecular species level annotation required the detection of fatty acyl chain-specific fragments. Lipids were filtered according to quality control criteria: signal-to-noise ratio greater than 10; “Peak Quality” index (calculated by LipidSearch software) greater than 0.7; m-score (calculated based on the number of matched product ion peaks in MS/MS spectrum) greater than 5; MS/MS data of each lipid species were detected in at least 50% of all samples. The MS/MS spectra was manually checked to confirm the identifications. Peak areas of lipid adducts were extracted for quantification, [M+H]^+^ and [M+NH_4_]^+^ for positive ionization mode, [M-H]^-^ and [M+HCOO]^-^ for negative ionization mode. To correct the signal variations during sample analysis, the peak area of each metabolite was normalized based on the QC samples using the quality control-random forest signal correction (QC-RFSC) method (4). After normalization, features with RSD >30% in QC samples were excluded. Finally, a total of 301 lipids with high quality were included with untargeted lipidomic analysis.

**Targeted lipidomics**

Targeted analysis of selected lipids was performed on a Vanquish™ UHPLC Systems coupled to TSQ Altis™ Triple Quadrupole Mass Spectrometer (Thermo Scientific, USA). Chromatography parameters were the same as our profiling method. The data was collected by using the selected reaction monitoring (SRM) mode. The spray voltage was optimized as 3.0 kV in both positive and negative ion mode. Calibrated RF lens which is automatically calculated by instrument according to the mass of each target ion was set. The pressure of sheath and auxiliary gas was set at 50 arb and 15 arb. The temperatures of the ion transfer tube and vaporizer were 285 °C and 300 °C respectively. The CID gas was set at 1.5 mTorr. The clinical samples were analyzed in a randomized order during UHPLC-MS/MS analysis.

The data process was performed by the TraceFinder Software (version 4.1, Thermo Scientific, USA). Lipids were quantified using the calibration curves of corresponding standards or structurally similar lipid species. Calibration was achieved by standard addition of selected lipid species or structurally similar lipid species to matrix samples containing isotope internal standards. The linearity was constructed by plotting the peak area ratio of each standard to internal standard against corresponding concentrations of standard with the application of a 1/X^2^ weighting factor. Intra-assay coefficients of variance showed values less than 20.0% for each lipid species, based on plasma quality control samples placed every 20 samples.

**Synthesis of diacylglycerol (DG) 18:2_22:6 and TG 12:0_18:2_22:6**

The procedure was adapted from a previous method by Halldorsson *et al* (5). Briefly, monolinoleate glycerol (100 mg, 0.282 mmol, Larodan, Sweden) and 4, 7, 10, 13, 16, 19-docosahexaenoic acid (100 mg, 0.304 mmol, Larodan, Sweden) were dissolved in 2 ml dichloromethane, followed by addition of 4-(dimethylamino)pyridine (DMAP) (30 mg, 0.24 mmol, Sigma-Aldrich) and N-(3-dimethylaminopropyl)-N’-ethylcarbodiimide hydrochloride (EDCI) (75 mg, 0.39 mmol, Sigma-Aldrich) into the solution. The mixture was then incubated for 1 hour with stirring. DG 18:2_22:6 then was purified by preparative high-performance LC.

DG 18:2_12:0 was obtained based on the same procedure using monolinoleate glycerol and lauric acid, DMAP and EDCI. Afterwards, 22:6 fatty acyl chain was added into DG 18:2_12:0 using 4, 7, 10, 13, 16, 19-docosahexaenoic acid in the presence of DMAP and EDCI in dichloromethane to give TG 12:0_18:2_22:6. The synthesized lipid species were validated by LC-MS/MS analysis. The chromatographic retention time and the MS/MS fragmentation spectra matched precisely with that of the corresponding lipidomic features.

**Mouse islet isolation**

C57BL/6N wild type mice fed on standard chow at 16 - 18 weeks of age were used. The animals were kept in cages in a 12h light-dark cycle with free access to water and diet. Mice fasted for 4 hours were subjected to in situ perfusion with 3 ml collagenase P (1.4 mg/ml, Roche) via the common bile duct, followed by subsequent digestion at 37°C for 15 min. The islets were then filtered through a 500-μm cell strainer and captured in a 70-μm cell strainer. The captured islets were washed with solution G (Hanks’ balanced salt solution, ThermoFisher Scientific, supplemented with 0.1% bovine serum albumin). The islets were incubated in RPMI 1640 supplemented with 10% FBS at 37°C in a humidified atmosphere containing 5% CO_2_ for 16-20 hours to recover. After that, islets with similar size were picked under a microscope and used for the glucose-stimulated insulin secretion assay.

**References**

1. Matyash V, Liebisch G, Kurzchalia TV, Shevchenko A, Schwudke D. Lipid extraction by methyl-tert-butyl ether for high-throughput lipidomics. J Lipid Res. 2008;49(5):1137-46.

2. Zhang H, Shao X, Zhao H, Li X, Wei J, Yang C, et al. Integration of Metabolomics and Lipidomics Reveals Metabolic Mechanisms of Triclosan-Induced Toxicity in Human Hepatocytes. Environ Sci Technol. 2019;53(9):5406-15.

3. Liebisch G, Fahy E, Aoki J, Dennis EA, Durand T, Ejsing CS, et al. Update on LIPID MAPS classification, nomenclature, and shorthand notation for MS-derived lipid structures. J Lipid Res. 2020;61(12):1539-55.

4. Luan H, Ji F, Chen Y, Cai Z. statTarget: A streamlined tool for signal drift correction and interpretations of quantitative mass spectrometry-based omics data. Anal Chim Acta. 2018;1036:66-72.

5. Halldorsson A, Magnusson CD, Haraldsson GG. Chemoenzymatic synthesis of structured triacylglycerols by highly regioselective acylation. Tetrahedron. 2003;59(46):9101-9.

**Fig. S1** **WGCNA of the lipid species profile. a** and **b**. Soft-thresholding powers of scale independence and mean connectivity from the analysis of network topology. Power 7 was chosen. **c.** Cluster dendrograms and module colors.


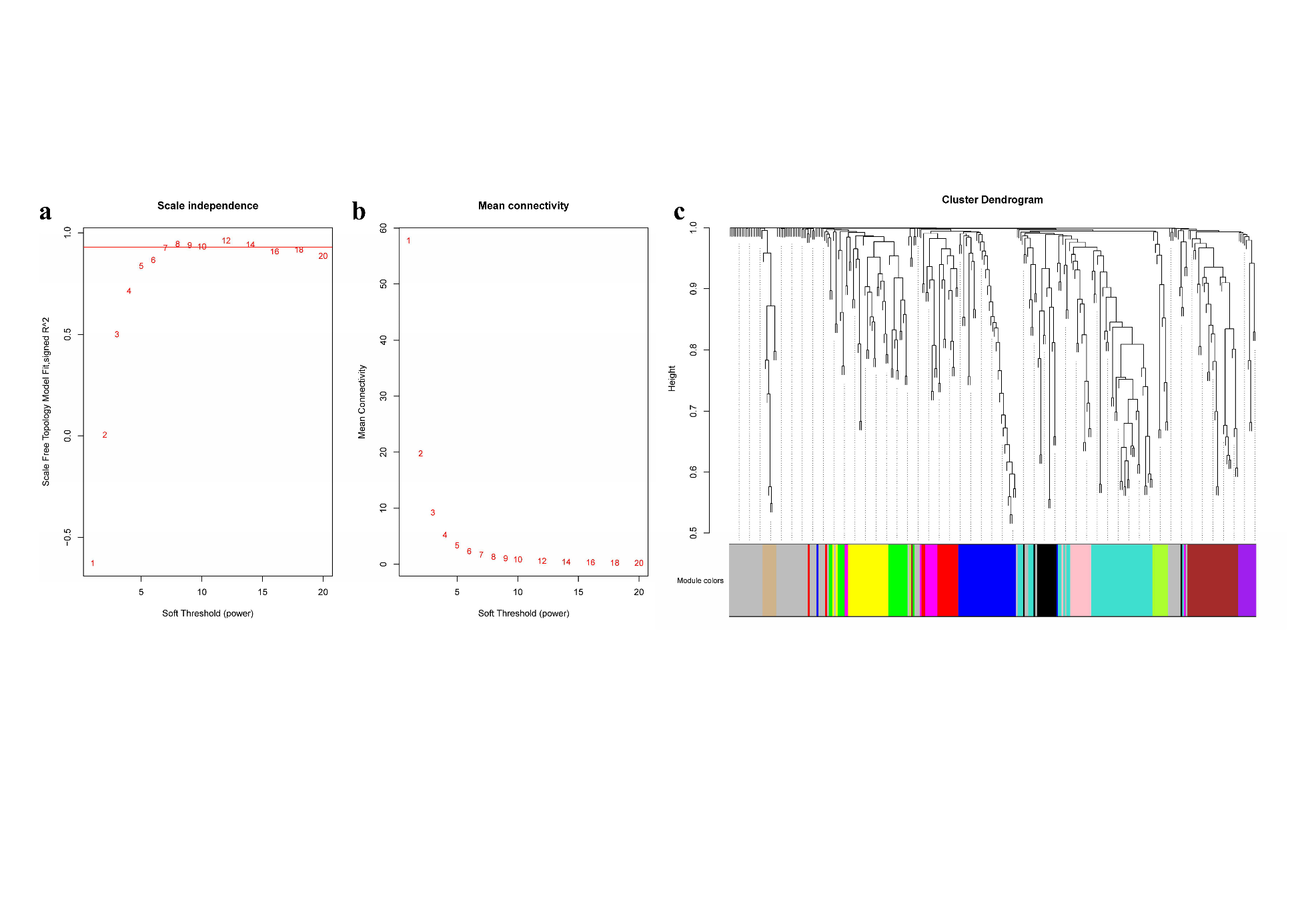


**Fig. S1d.** Correlation between the modules and clinical traits. Module blue, red, green, yellow, pink, greenyellow, black, turquoise, brown were significantly correlated with incident type 2 diabetes. **e.** The components of identified modules.


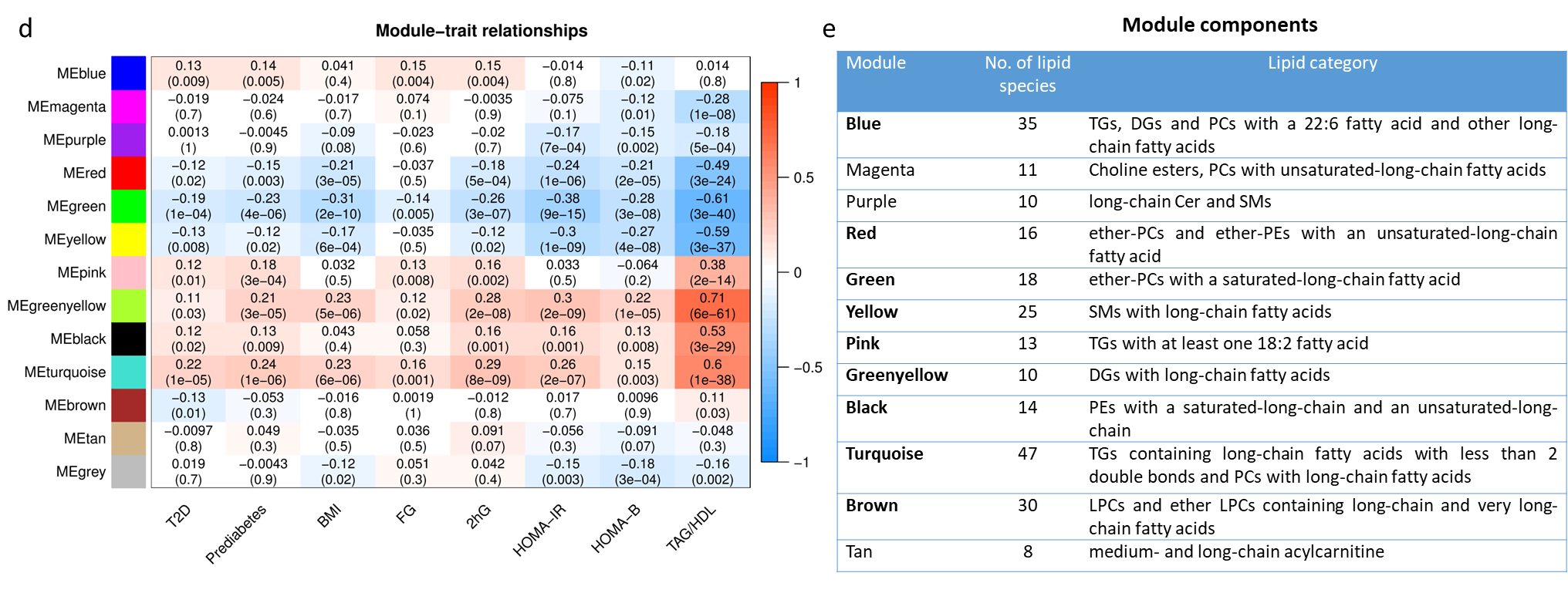


**Fig. S1f.** The constructed network of the important modules. A total of 163 nodes were included in the network with a connectivity threshold of 0.1. The bolded lipid species were those also identified in the Boruta analysis.


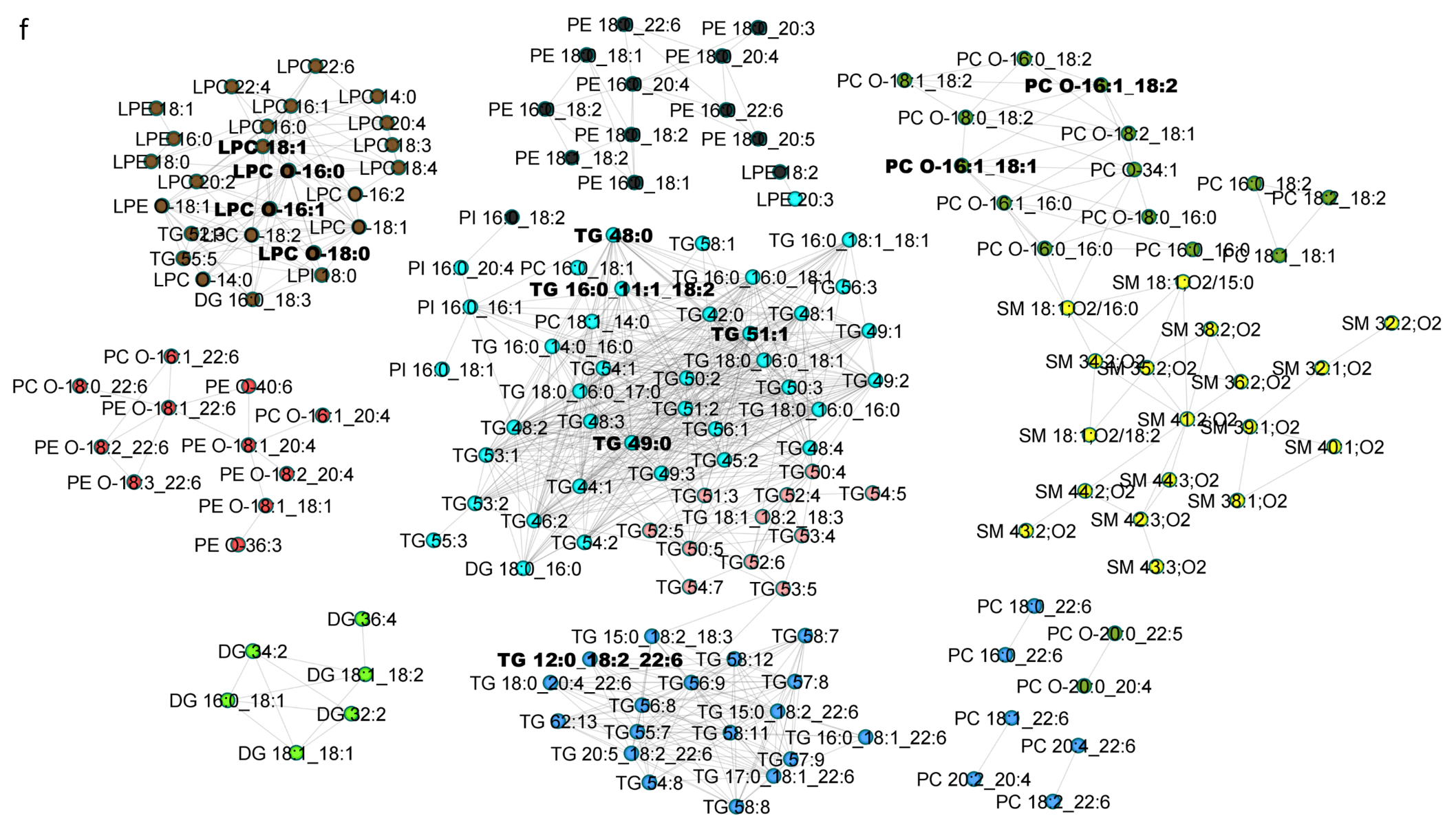


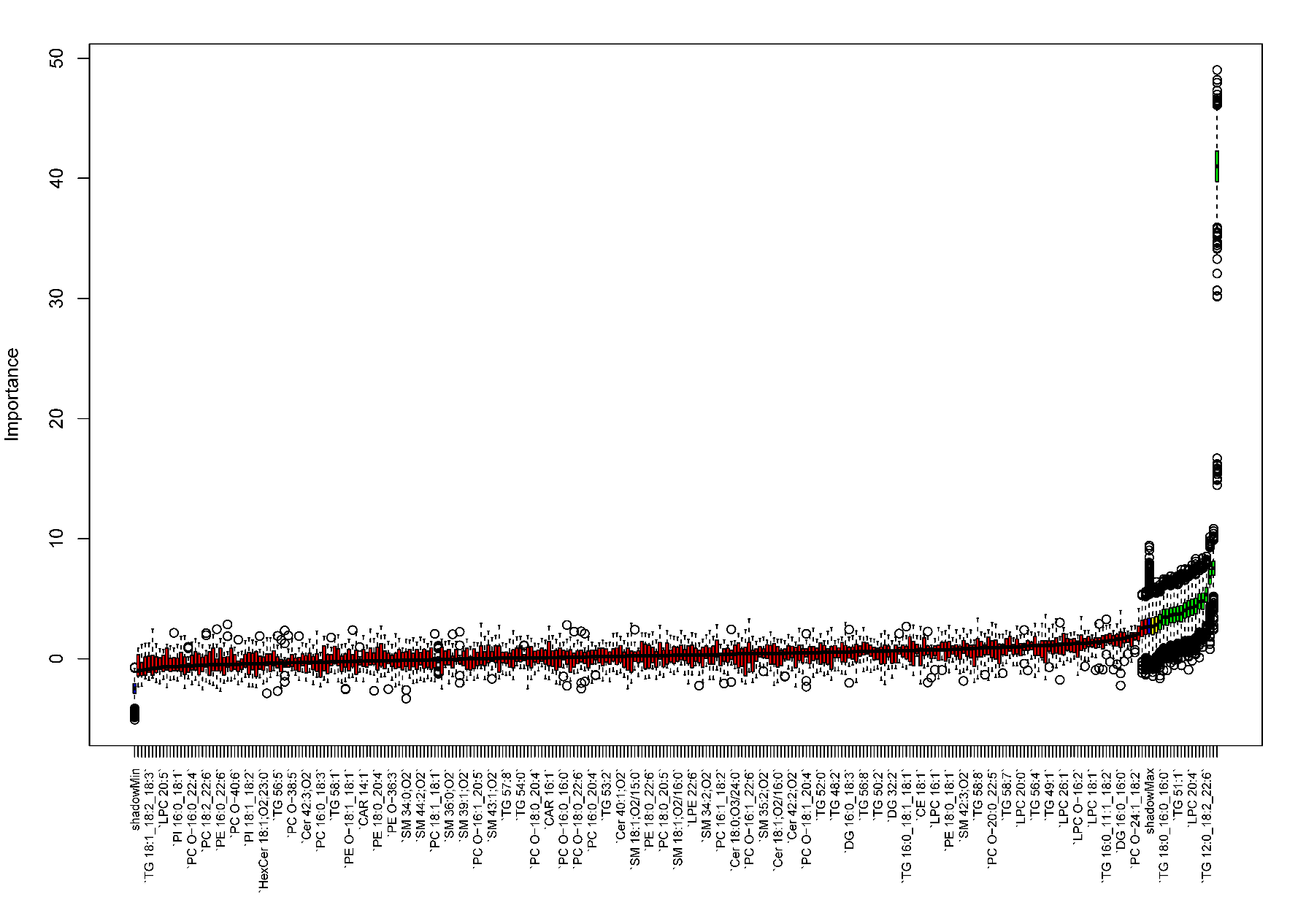
**Fig. S2 Boruta analysis result.** Lipid species were compared with algorithm-generated shadow features. The green and red boxes represent the importance z scores of confirmed and rejected lipid species, respectively. The blue boxes are the shadow attributes. A total of 16 lipid species were confirmed.


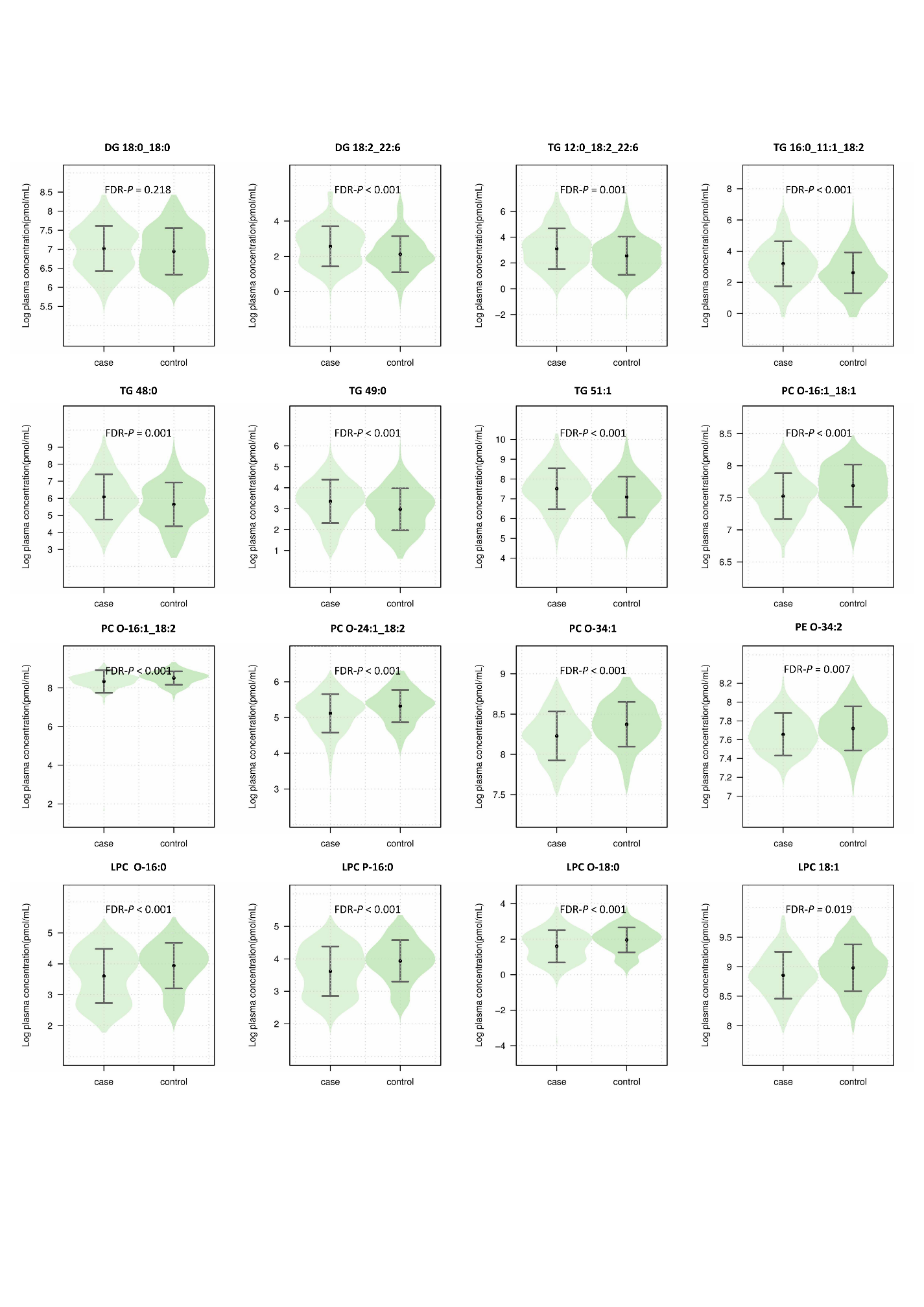
**Fig. S3 Violin Plot of the Targeted Lipidomics.** All of the lipid species quantified by the targeted lipidomic analysis, except DG 18:0_18:0, were significantly different between the incident type 2 diabetes cases and non-type 2 diabetes controls (all unadjusted p <0.01; *q*-value < 0.10).

**Fig. S4 Correlation among identified lipid species.** A correlation heat map illuastrating the correlation among the 16 lipid species identified by Boruta Analysis. Positive and negative correlations are shown in blue and red, respectively, with digits indicating the correlation coefficients.


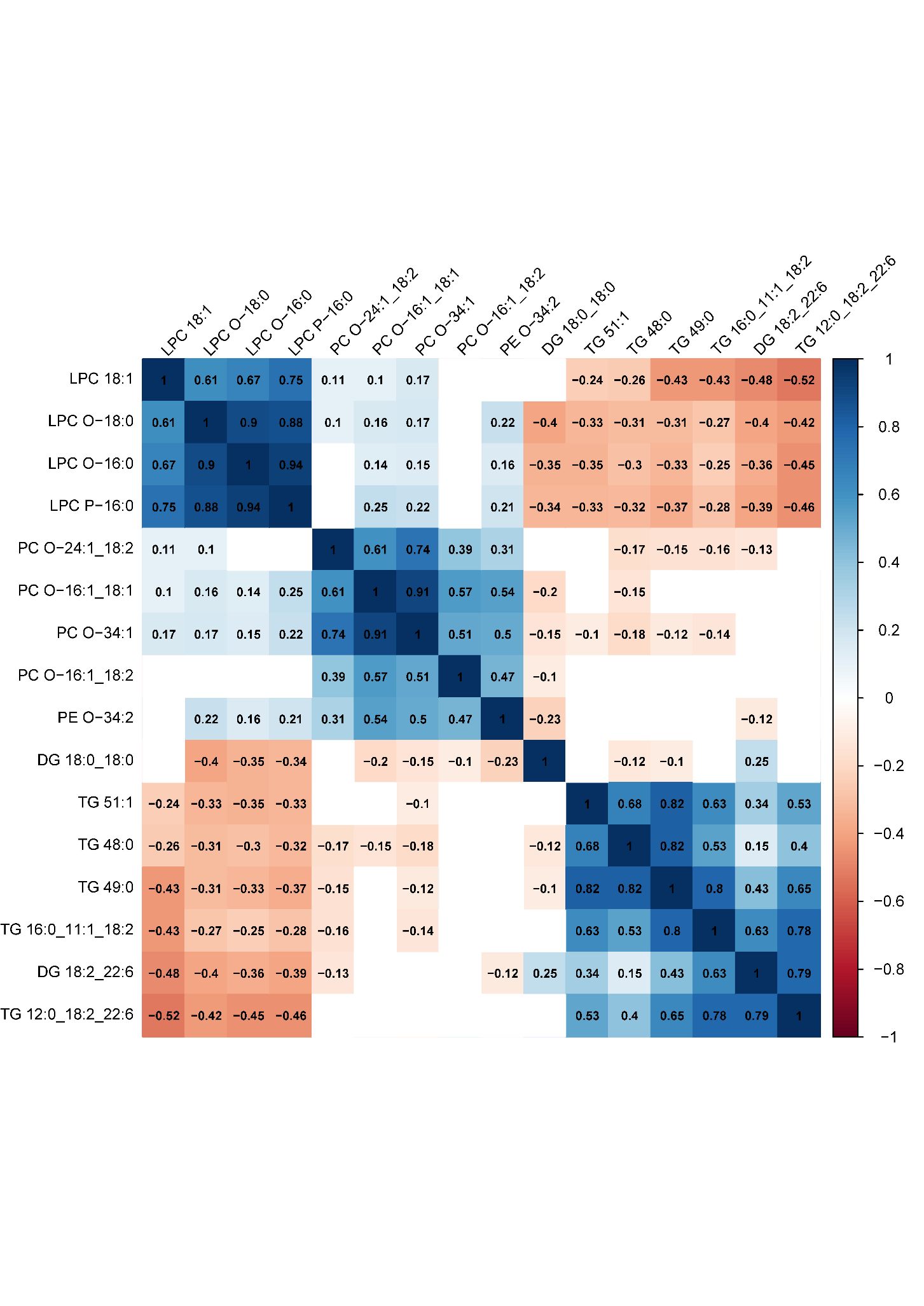

Supplement: Supplementary file 1 — Additional file 1. Methods. Figure S1. WGCNA of the lipid species profile. Figure S2. Boruta analysis result. Figure S3. Violin Plot of the Targeted Lipidomics. Figure S4. Correlation among identified lipid species. [file 12933_2022_1677_MOESM1_ESM.docx]
